# Prognosis impact and clinical findings in renal cancer patients: comparative analysis between public and private health coverage in a cross-sectional and multicenter context

**DOI:** 10.1007/s10552-024-01891-3

**Published:** 2024-11-08

**Authors:** Eduardo Barrera-Juarez, Antonio Nassim Halun-Trevino, Manuel Ruelas-Martinez, Andres Madero-Frech, Victor Camacho-Trejo, Miguel Estrada-Bujanos, David Bojorquez, Jhonatan Uribe-Montoya, Francisco Rodriguez-Covarrubias, Cynthia Villarreal-Garza

**Affiliations:** 1 Tecnologico de Monterrey, Escuela de Medicina y Ciencias de la Salud, Ave. Morones Prieto 3000, 64710 Monterrey, Nuevo Leon Mexico; 2 Hospital Metropolitano Servicios de Salud Nuevo Leon, Monterrey, Mexico; 3 Instituto Mexicano del Seguro Social, Monterrey, Mexico; 4 Instituto de Seguridad y Servicios Sociales de los Trabajadores del Estado, Monterrey, Mexico; 5 Instituto Nacional de Ciencias Medicas y Nutricion “Salvador Zubiran”, Mexico City, Mexico; 6 Clinical Oncology Chairman Tecnologico de Monterrey, Monterrey, Mexico

**Keywords:** Renal tumor, Tumor size, Advanced disease, Public coverage, Health disparities

## Abstract

**Purpose:**

Research on disparities in prognosis and clinical characteristics between public and private healthcare sectors in developing countries remains limited. The study aimed to determine whether patients with public health coverage (1) have a greater mean tumor size at diagnosis compared to those with private health coverage; (2) exhibit differences in clinical staging and TNM classification between groups; and (3) show variations in demographic, clinical characteristics, histopathological findings, and surgical approaches among cohorts.

**Methods:**

A cross-sectional, multicenter study was conducted on 629 patients from both private and public healthcare sectors, all histologically confirmed and surgically treated for Renal Cell Carcinoma (RCC), between 2011 and 2021 in high-volume hospitals in Monterrey, Mexico. To compare variables between groups, we employed independent samples *t*-tests, Mann Whitney *U* nonparametric test, along with Pearson’s chi-square test complemented by post hoc analyses.

**Results:**

Mean tumor size in the public group was 1.9 cm greater than in the private group (7.39 vs. 5.51 cm, *p* < 0.001). Patients in the public sector more frequently presented with larger tumors, a higher prevalence of risk factors (excluding BMI and hypertension), advanced disease (OR 2.12, 95% CI 1.43–3.16, *p* < 0.001), presence of symptoms, elevated TNM, lymphovascular invasion and a lower prevalence of minimally invasive surgery. A male-to-female ratio of 2.6:1 was noted in the private coverage group.

**Conclusions:**

This study highlights a notable association between public health coverage and a higher prevalence of advanced RCC, with tumors in private coverage patients being smaller yet larger than commonly reported. There is a crucial need to develop new health policies for early detection of renal cancer in developing countries.

**Supplementary Information:**

The online version contains supplementary material available at 10.1007/s10552-024-01891-3.

## Background

Radical or nephron-preserving surgery is the only curative treatment for renal cancer [1]. RCC cannot be cured if it escapes the time window for surgical treatment and persists in the patient with local or distant invasion [2]. Thus, renal cancer can be deadly if not detected before the development of clinical symptoms [3–5], and it remains a highly lethal urologic tumor. Currently, around 17–30% of patients have distant metastasis at diagnosis, with the most commonly affected sites being the lungs (71%), lymph nodes (49%), bone (36%), and liver (21%) [6]. Renal cancer is the sixth most common cancer in males and the tenth in females, corresponding to 5% and 3% of all oncological diagnoses, respectively. Recently, the stage of smaller and localized tumors at diagnosis has increased due to improved diagnostic tools; however, this is not a global reality. Patients from developing countries have more extensive and advanced tumors compared with patients from countries with higher economic development [7–9].

One of the primary challenges in diagnosing renal cancer lies in its asymptomatic progression, which can delay detection until advanced stages. Only 10% of patients present with the classical triad of hematuria, flank pain, and a palpable mass, and these are only noted when the tumor has reached a considerable size [10, 11]. Smaller tumors can present with a benign history and can be removed entirely with surgery or minimally invasive treatments [12, 13]. Size has been reported as an independent risk for renal carcinoma, and larger sizes increase dissemination and mortality risk among patients, particularly in the advanced stages [14–16]. Such malignancies are identified incidentally in a mere subset of patients. The challenge is to extend the identification opportunity to more vulnerable and unprotected groups [17].

In Mexico, the health system is complex and includes public and private health service providers. The public health system lacks sufficient funds and personnel, leading to long waiting times and limited access to health care. Significant inequality has been observed between the public and private health sectors regarding stage, prognosis, and mortality in different types of cancers [18–20]. However, there is limited information about this disparity for renal cancer. Monterrey, located in northern Mexico in the state of Nuevo Leon, is the third-largest city in Mexico in terms of economic contribution. It is an industrial and cosmopolitan city, with 96% of the population in urban areas and 4% in rural areas [21]. Approximately 81.5% of residents have access to public health systems, about 16.5% to private health systems, and 1.9% have unrecognized health coverage [22].

This study aims to determine whether patients with public health coverage have a greater mean tumor size at diagnosis compared to those with private health coverage, and to evaluate if there are differences in clinical staging and The American Joint Committee on Cancer (AJCC) tumor-node-metastasis (TNM) 8th edition classification between these groups. Additionally, the study appraises demographic, clinical characteristics, histopathological findings, and surgical approaches among cohorts.

## Methods

### Data source

This observational, cross-sectional, multicenter study included patients with a pathological diagnosis of renal cancer who underwent surgical treatment in high-volume hospitals in Monterrey, Mexico. Three institutions represented the public health coverage group: (1) *Hospital de Alta Especialidad*, IMSS Unit 25 of the *Instituto Mexicano del Seguro Social*, (2) *ISSSTE Regional* from the *Instituto de Seguridad y Servicios Social de los Trabajadores del Estado de Nuevo León*, and (3) *Hospital Metropolitano de Monterrey*. The private health coverage was represented by three prestigious establishments such as (1) *Hospital Zambrano-Hellion, *(2) *Hospital San Jose TECSalud, and *(3) *Hospital Angeles Valle Oriente.*

After receiving approval from the Research Ethics Review Committee, the data were collected by reviewing the patients’ medical records. All patients were treated surgically and had a diagnosis of primary renal cancer (pathological study and classification). The evaluation period spanned from 2011 to 2021.

### Study population and variables definition

The inclusion criteria were the following: patients of any age with a radiological diagnosis (computed tomography [CT] or magnetic resonance [MR]) of primary renal cancer, treatment with radical nephrectomy or nephron-preserving surgery, and a proper staging and pathological diagnosis of renal cell carcinoma (RCC) classified according to AJCC TNM 8th edition classification. Patients with incomplete chart information on pathological diagnosis or without radical or partial surgical treatment were excluded.

Clinical, pathological, and demographic variables analyzed included age, tumor size based on histopathological size (cm), and body mass index (BMI) as continuous variables. Nominal and ordinal variables were: gender, tumor focus (single or multiple), surgical approach (conventional or minimally invasive), type of surgery (radical or nephron-sparing), clinical presentation (hematuria, palpable mass, pain, other symptoms, or asymptomatic), risk factors (diabetes mellitus, obesity, smoking, and systemic hypertension), lymphovascular invasion, tumor pathological stage according to the TNM, clinical stage (I, II, III, IV), localized or advanced-stage RCC according on American Joint Committee on Cancer staging system (clinical stage I, II vs III, IV) [33], tumor size greater or less than 7 cm, and histological type.

### Sample size calculation

The formula used to calculate the sample size for a two-sample difference of means test with an alpha of 0.05 and power of 80% was as follows:

$n = \left( {{{Z\alpha } \mathord{\left/ {\vphantom {{Z\alpha } 2}} \right. \kern-0pt} 2} + Z\beta } \right)^{2} \times \sigma 1^{2} /\Delta^{2} + \left( {{{Z\alpha } \mathord{\left/ {\vphantom {{Z\alpha } 2}} \right. \kern-0pt} 2} + Z\beta } \right)^{2} \times {{\sigma 2^{2} } \mathord{\left/ {\vphantom {{\sigma 2^{2} } {\Delta^{2} \times k^{2} }}} \right. \kern-0pt} {\Delta^{2} \times k^{2} }}$ where Δ =|*μ*2 − *μ*1| = absolute difference between two group means; *σ*1, *σ*2 = variance of mean #1 and mean #2; *n*1 = sample size for group #1; *n*2 = sample size for group #2; *α* = probability of type I error; *β* = probability of type II error; *Z* = critical *Z* value for *α* or *β*; and *k* = proportion of the sample size for group #2 to group #1.

We considered 186 patients for each group.

### Statistical analysis

Continuous variables were expressed as mean ± standard deviation, median, maximum, minimum, and 95% confidence interval. Categorical variables were presented as absolute and relative frequencies. The Student’s *t*-test was used for parametric continuous variables, and the Mann–Whitney *U* test for nonparametric continuous variables. The chi-squared test was used for categorical variables complemented with post hoc analyses. Patient charts that lacked tumor size information were excluded. For other continuous and categorical variables, cases with missing data were excluded from the bivariate analysis. All statistical analyses were conducted using SPSS version 29.0 (IBM Corp., Armonk, NY), with the significance level set at 0.05.

## Results

The clinical records of 636 patients were reviewed, seven patients had to be removed due to lack of information, resulting in a final cohort of 629 patients for the analysis. The sample mean age was 59 years (11–95 years), with no statistical difference between groups. (*p* = 0.71) Among these 629 patients, 368 (65.5%) were included in the public coverage group and 217 (34.5%) in the private group. The overall gender distribution was 389 (61.81%) male patients (233 [56.6%] in the public group and 156 [71.9%] in the private group), and 240 (38.15%) female patients (179 [43.4%] in the public group and 61 [28.1%] in the private group). Notably, in the private group, there was a male-to-female ratio of 2.6:1 (Table 1).

**Table 1:** Demographic characteristics across public and private health coverage groups

		Public Healthcare	Private Healthcare	*p*
		*N *(%)		
	*N* 629	412 (65.5)	217 (34.5)	
Age in years		59	59	0.7
Male		233 (56.5)	156 (71.8)	< 0.001**
Female		179 (43.4)	61 (28.1)	< 0.001**
DM	*n* 619	130 (31.5)	44 (21.2)	0.007*
Tobacco	*n* 617	131 (31.9)	40 (19.4)	< 0.001**
HTS	*n* 619	187 (45.3)	77 (37.2)	0.52
(BMI > 30) obesity	*n* 597	171 (44.7)	47 (22.7)	< 0.001**
BMI	*n* 466	29.35	29.77	0.5
Height (mts)	*n* 490	1.63	1.71	< 0.001**
Weight (kg)	*n* 490	77.71	87.39	< 0.001**

*Comparative analysis of tumor sizes across diverse coverage groups*				
		Public Healthcare	Private Healthcare	*p*
	*N 629*	*N 412*	*N 217*	
		cm	cm	
Mean		7.39	5.51	< 0.001**
Median		7	5	
SD		3.9	2.97	
Minimum		0.6	0.1	
Maximum		30	15	
95% CI		7.01–7.77	5.12–5.91	
		*N (%)*	*N (%)*	
< 7 cm		219 (53.2)	167 (23)	< 0.001**^a^
> 7 cm		193 (46.8)	50 (77)	

*DM* diabetes mellitus, *HTS* hypertension, *BMI* Body Mass Index, *mts* meters, *kg* kilograms, *SD* standard deviation, *CI* confidence interval

*p value < 0.05

**p value < 0.001

^a^Odds ratio 2.94 (CI 95% 2.03–4.26)

Discrepancies were observed between groups related to risk factors, such as tobacco use (32.0% vs. 19.3%, *p* < 0.001), presence of diabetes mellitus (31.6% vs. 21.3%, *p* = 0.007), and obesity (BMI greater than 30 kg/m^2^, *p* < 0.001). However, no significant differences were observed in the BMI and hypertension across groups. The mean pathological tumor size in the public coverage group was more than 1.88 cm greater than that in the private coverage group (7.39 cm vs. 5.51 cm, *p* < 0.001) (Table 1). 46.8% of patients in the public coverage group had tumors larger than 7 cm compared to 23% in the private group (OR 2.94 95% CI 2.03–4.26 *p* < 0.001).

**Fig. 1:**
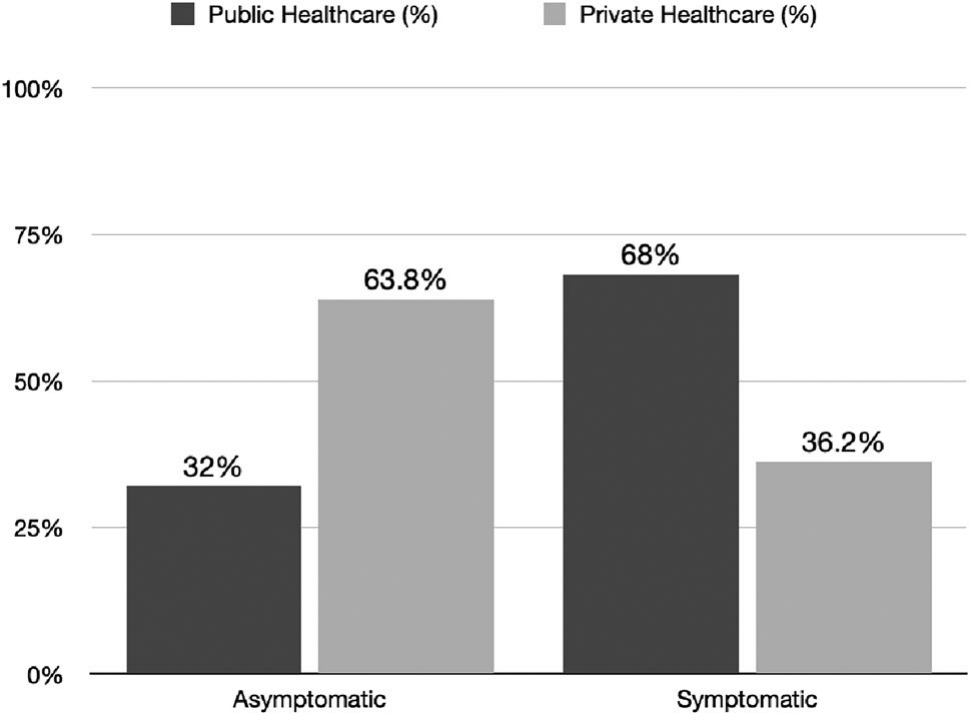
Asymptomatic and symptomatic patients in both health coverage groups

Our clinical research study collected symptoms data from a total of 629 patients. Unfortunately, it was not possible to collect information on the symptoms of 51 people in the study population. Of these, 261 (54.8%) were asymptomatic, and a symptomatic presentation was noted in 317 (45.2%). We identified 131 (76.8%) asymptomatic patients in the private insurance group compared to 130 (32%) in the public group. In contrast, there were 41 (23.8%) symptomatic patients in the private group compared to 276 (68%) in the public group. (Fig. 1). In an in-depth analysis of symptoms, we found hematuria in 167 patients (27.5%) (21.2% derived from public coverage vs. 6.3% among the private group), flank pain in 183 patients (30%) (24.2% derived from public coverage vs. 5.8% among the private group), and finally palpable mass in only 24 patients (0.39%) All groups had *p* < 0.001 (Table 2).

**Table 2:** Comparative analysis of symptomatology among patients with public and private health coverage

	Public	Private	*n*	*p* value
	Frequency *n* (%)			
Hematuria	129 (21.2)	38 (6.2)	167 (27.5)	< 0.001**
Flank pain	147 (24.2)	36 (5.8)	183 (30.1)	< 0.001**
Palpable mass	23 (0.38)	1 (0.002)	24 (3.9)	< 0.001**

(*n* = 608)

*p value < 0.05

**p value < 0.001

Of the 629 patients, clear cell RCC (ccRCC) was the predominant histological type represented in 537 patients (85.4%), followed by Papillary RCC in 40 (6.4%) and Chromophobe RCC in 24 cases (3.8%). The rest included Mucinous tubular, MIT Family Translocation-associated RCC and Collecting duct carcinoma (Table 3).

**Table 3:** Histopathological outcomes of patients across both public and private health coverage groups

	Public	Private	*p*
	Frequency *N* (%)		
*n* 629	412 (65.5)	217 (34.5)	
RCC	366 (88.8)	171 (78.8)	< 0.001**
Papillary RCC	21 (5.1)	19 (8.8)	0.074
Chromophobe RCC	11 (2.7)	13 (6.0)	0.039*
Cystic multilocular	7 (1.7)	3 (1.4)	0.763
Others^a^	7 (1.7)	11 (5.1)	0.016*

*RCC* renal cell carcinoma

*p value < 0.05

**p value < 0.001

^a^Others include Mucinous tubular, MIT Family Translocation-associated RCC, Collecting duct carcinoma

When the tumors were classified according to the TNM AJCC 8th edition staging system, significant differences were noted between the coverage groups. Smaller and localized tumors were noted in the private healthcare group compared to public patients. (Table 4) This difference was significant in the T1a (21.4% in the public healthcare group vs 37.2% in private patients, *p* < 0.001), T2a (18.2% vs 11.6%, *p* = 0.033) and T2b (10% vs 3.7%, *p* = 0.006) classification. Notably, no significant difference was found in higher staged-tumors. The prevalence of lymph node invasion was higher in the public coverage group compared to the private group (N1: 10.2% vs. 3.7%, *p* < 0.005), a similar trend was observed for metastasis, with the public group showing a higher rate (M1: 13.4% vs. 7.5%, *p* = 0.029).

**Table 4:** Clinical and pathological staging comparison between private and public healthcare groups in renal cancer: AJCC TNM 8th Edition Classification, and TNM stage grouping

AJCC TNM		Public	Private	*p*
		Frequency *N* (%)		
T	*N 627*	412. (65.5)	215 (34.5)	
T1a		88 (21.4)	80 (37.2)	< 0.001**
T1b		10 (25.0)	66 (30.7)	0.127
T2a		75 (18.2)	25 (11.6)	0.033*
T2b		41 (10.0)	8 (3.7)	0.006*
T3a		65 (15.8)	23 (10.7)	0.082
T3b		13 (3.2)	6 (2.8)	0.800
T3c		5 (1.2)	0	0.105
T4		22 (5.3)	7 (3.3)	0.238
N	*N 627*	412 (65.5)	215 (34.5)	
Nx		0	3 (1.4)	0.016*
N0		370 (89.8)	204 (94.9)	0.030*
N1		42 (10.2)	8 (3.7)	0.005*
M	*N 624*	411 (65.7)	213 (34.3)	
M0		356 (86.6)	197 (92.5)	0.029*
M1		55 (13.4)	16 (7.5)	
Clinical stage				
*n *626				
I		183 (44.6)	143 (66.5)	< 0.001**
II		91 (22.2)	31 (14.4)	0.021*
III		70 (17.1)	21 (9.8)	0.014*
IV		67 (16.1)	20 (9.3)	0.016*
Localized stage (I/II) vs advanced stage (III/IV)				
*N 626*				
Localized		274 (66.7)	174 (80.9)	< 0.001**^a^
Advanced		137 (33.3)	41 (19.1)	

*p value < 0.05

**p value < 0.001

^a^Odds ratio 2.12 (CI 95% 1.43–3.16)

Clinical staging according to the AJCC TNM stage groups was employed for 625 patients with complete information and demonstrated a significant difference (*p* < 0.001) between the groups. More advanced grades were in the public coverage group than the private group. Stage I accounted for 326 (52.1%) patients (183 [44.6%] for the public group versus 143 [65.5%] for the private group), stage II corresponded for 122 (19.5%) patients (91 [22.2%] for the public group versus 31 [14.4%] for the private group), stage III comprised 91 (14.5%) patients (70 [17.1%] for the public group versus 21 [9.8%] for the private group), stage IV was represented by 86 (13.8%) patients (66 [16.1%] for the public group compared to 20 [9.3%] for the private group). 71.6% of patients had localized tumors (CS I, II), while 28.4% had advanced tumors (CS III, IV) (OR 2.12, IC 95% 1.43–3.16, *p* < 0.001) (Table 4).

**Fig. 2:**
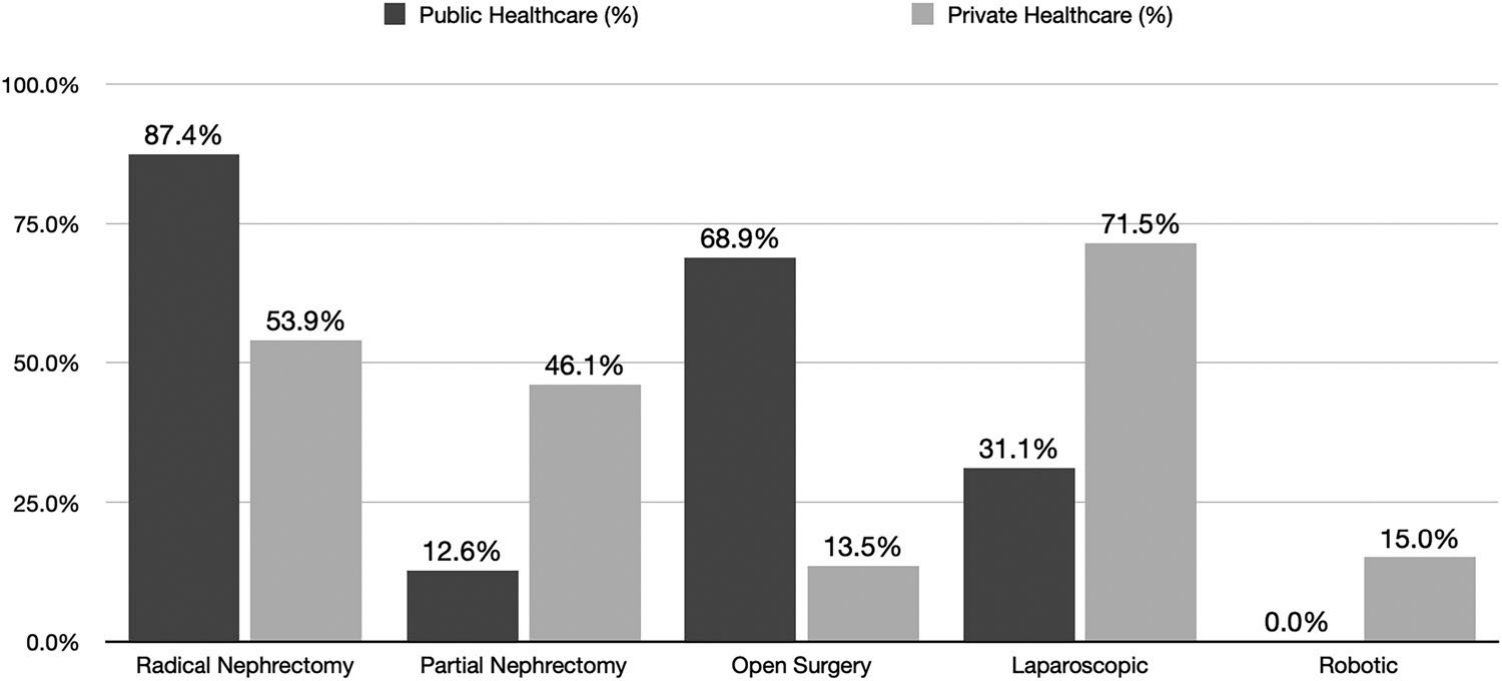
Surgical approaches between groups

Radical nephrectomy was performed more frequently in the public compared to the private group (360 [87.4%] vs. 117 [54%] *p* < 0.001), open surgery was more prevalent in the public coverage group than in the private coverage group (283 [68.9%] vs. 27 [13.54%] *p* < 0.001). Conversely, laparoscopic surgeries were more common in the private coverage group (128 [31.1%] public vs. private 143 [71.5%] *p* < 0.001) (Fig. 2). Robotic surgery was exclusively performed on patients within the private coverage.

## Discussion

### Main findings

We observed significant differences in outcomes between patients with public and private insurance. Renal tumors tended to be more advanced and larger in the public compared to the private insured. Patients with a higher prevalence of risk factors were also more likely to be in the public sector (excluding BMI and hypertension), to have advanced tumors (OR 2.12), to be symptomatic, to have increased TNM, and to undergo less minimally invasive surgery.

These results indicate that there is an equity issue regarding access to medical care among patients with renal tumors. Patients with public coverage could encounter barriers when seeking out healthcare services that could help detect and treat tumors at an earlier stage. Furthermore, the results can help identify areas that require further investigation to understand the differences better in medical care access between those with public and private coverage, as well as develop interventions, policies, and healthcare programs that might aid in narrowing these differences and provide all patients with access to high-quality healthcare.

For oncologic pathologies, it is well known that there are social inequalities regarding healthcare, timely detection, and treatment [23–25, 33]. In our study, this phenomenon was evident. Notably, even among the private coverage patients, who generally have a greater opportunity for early diagnosis, the average tumor size was not as small as typically reported in the literature [26–28]. Additionally, in our cohort, a significant discrepancy was observed in the prevalence of asymptomatic tumor discovery, with 76.2% of tumors in the private group being found incidentally, compared to only 32% in the public group. This highlights the disparities in diagnostic practices and patient awareness between the two healthcare sectors.

The Latin American Renal Cancer Group (LARCG) reported a significant hazard ratio (HR 1.64) for worsened 10-year overall survival in renal cancer patients with tumors larger than 7 cm, noting such tumor sizes in 27.7% of their patients. In contrast, our cohort demonstrated a notably higher likelihood of tumors > 7 cm in the public sector (46.8% vs 23% in the private sector) with an OR of 2.94 (95% CI 2.03–4.26, *p* < 0.001) [29]. This underscores the critical impact of healthcare access disparities on tumor progression and patient outcomes. Therefore, we must consider the importance of detecting clear cell renal tumors in asymptomatic patients and in the assessment of other pathologies. It has been reported that the detection of renal tumors is increasing, with smaller tumors being found [8, 30, 31]; however, as stated, this is not always the case.

As healthcare personnel, we must rethink the incidence and prevalence of regional renal tumors. Do we genuinely observe a rise in the detection of smaller renal tumors through campaigns, early detection methods, and imaging tools, or could we be merely inclined to perceive an increase in the incidence of smaller renal tumors owing to our reliance on the literature? The assessment should consider the future of current patients with renal tumors and not lose sight of the fact that those with smaller tumors have a greater chance of survival.

The International Agency for Research on Cancer of the World Health Organization reported an incidence of 26/per 100,000 in Latin America, with an estimated increase of 62% by 2040 [32]. For Mexico, an increase of 107% is estimated by 2040 [32]. Will the majority of these future tumors be discovered as small asymptomatic masses? It is undisputed that the best hope for a patient having renal cancer is for it to be small enough to avoid cellular dissemination that cannot be managed with current therapeutic measures. Studies are needed to identify the best way to detect smaller tumors susceptible to initial treatments that can be successfully managed.

### Limitations

The study has some limitations. Most importantly the inherent bias as a nonrandomized retrospective study. The identification of patients with more advanced tumors, which are not amenable to surgery, was impaired by the lack of comprehensive surveillance and incidence data. This shortcoming led to an implicit omission bias. However, to achieve external power in the study, patients from high-volume public and private hospitals were included, and the study represents at least 30% of the population.

## Conclusions

This study reveals a significant association between public health coverage and higher prevalence of extensive and advanced RCC, with a 2.12-fold increase in the likelihood of more advanced RCC in the public health sector. Although tumor sizes were smaller in patients with private coverage, they were not as small as commonly reported in the literature. This highlights the urgent need for the early identification of asymptomatic renal cancer patients across all healthcare settings. The establishment of regional, accurate surveillance and epidemiological data on renal tumors is crucial for a detailed assessment of cancer incidence and prevalence, ultimately aiming to reduce mortality rates. Reforming health policies and creating regional guidelines specifically for renal cancer are imperative steps towards this goal. Additionally, increasing the detection of new and smaller tumors remains a priority. Furthermore, our findings suggest a lower incidence of renal cancer in women under private health programs compared to their counterparts in public health coverage. Investigating the underlying reasons for this discrepancy is vital for developing more effective and equitable cancer care strategies.

## Supplementary Information

Below is the link to the electronic supplementary material.

Supplementary file1 (PNG 189 KB)

Supplementary file2 (PNG 144 KB)

## Data Availability

Dataset generated and analyzed during the current study are not publicly available due to non-disclosure agreements and privacy policies of the participating hospitals, which prohibits public sharing of patient data, but are available from the corresponding author on reasonable request.
