# Supplementary figures and images for: Prognosis impact and clinical findings in renal cancer patients: comparative analysis between public and private health coverage in a cross-sectional and multicenter context
Source: Cancer Causes Control. 2024 Nov 8;36(3):265–73. doi: 10.1007/s10552-024-01891-3 (PMC11928398; doi:10.1007/s10552-024-01891-3)

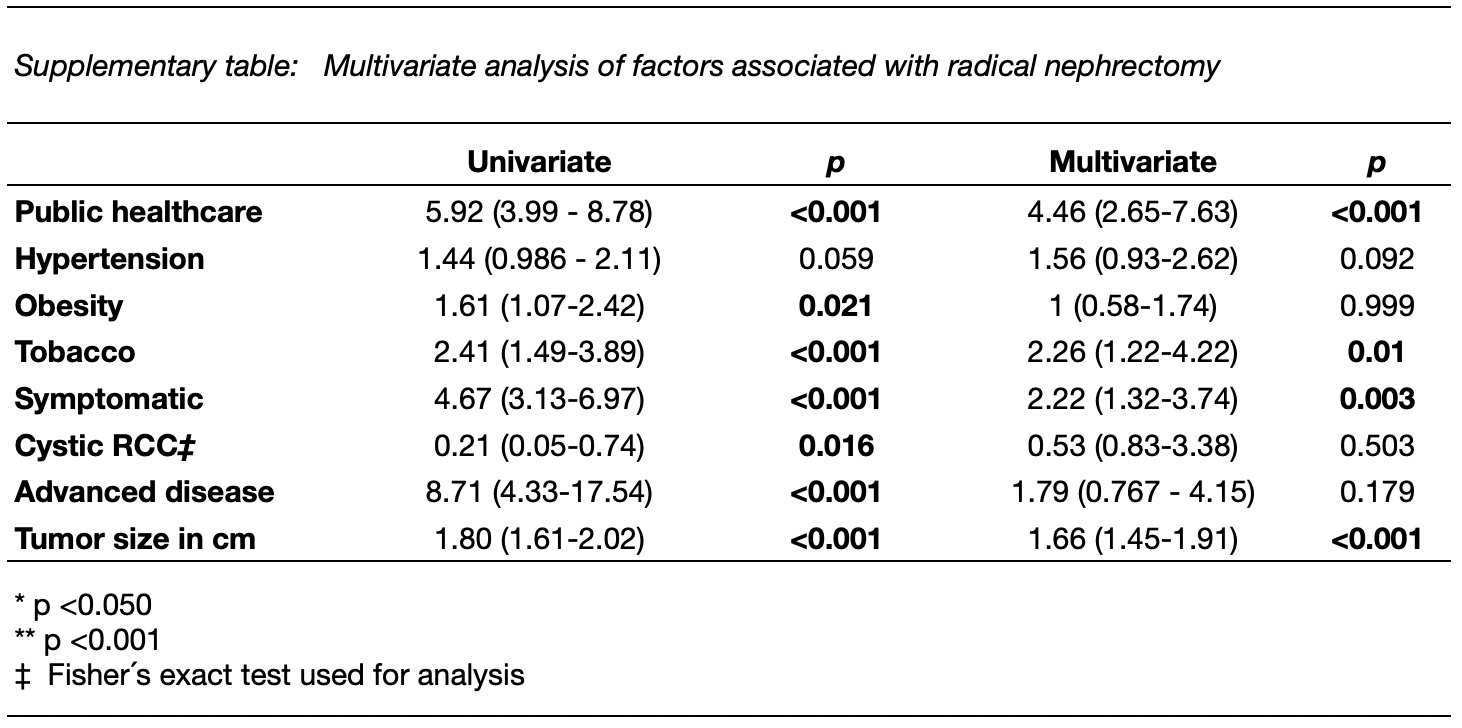

Supplement: Supplementary file 1 — Supplementary file1 (PNG 189 KB) [file 10552_2024_1891_MOESM1_ESM.png]

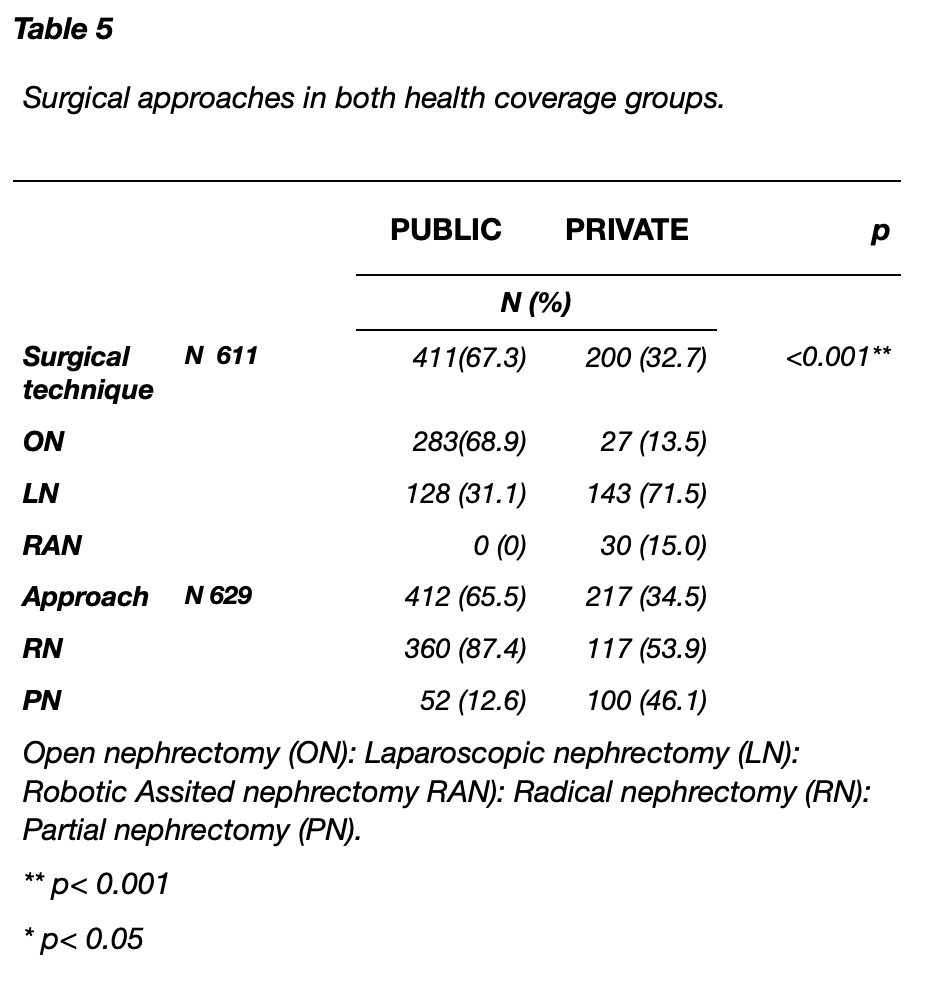

Supplement: Supplementary file 2 — Supplementary file2 (PNG 144 KB) [file 10552_2024_1891_MOESM2_ESM.png]
